# Radiation doses during computed tomography scan imaging in Hassan II Hospital, Agadir, Morocco

**DOI:** 10.11604/pamj.2022.41.290.25090

**Published:** 2022-04-11

**Authors:** Slimane Semghouli, Bouchra Amaoui, Soukaina Wakrim

**Affiliations:** 1 Higher Institute of Nursing Professions and Health Techniques, Agadir, Morocco,; 2 Faculty of Medicine and Pharmacy, Ibn Zohr University of Agadir, Agadir, Morocco

**Keywords:** Computed tomography scan, diagnostic reference levels, assessment, radiation risks, Moroccan hospital

## Abstract

**Introduction:**

the aim of this study was to establish local Diagnostic Reference Levels (DRLs) for four adult Computed Tomography (CT) examinations in the radiology department, Hassan II hospital of Agadir.

**Methods:**

during this survey, we have examined the data of 200 patients at an average of 50 per localization. A General Electric 16 CT with automatic exposure control system was used to perform all CT examinations. Scanner acquisition parameters, number of series, contrast medium use, rotation time plus slice thickness, the displayed Computed Tomography Dose Index (CTDI_vol_), and the Dose Length Product (DLP) were among the data collected for each diagnostic exam chosen. To evaluate the DRL and effective dose, the conversion factor and formalism of the International Commission on Radiological Protection (ICRP) were utilized.

**Results:**

the average effective dose (Eeff), the displayed CT dose index (CTDI_vol_), dose length product (DLP) were (7.28±2.35) mSv, (10.80±3.80) mGy and (428.35±138.26) mGy.cm respectively at chest CT. For abdomen-pelvis CT scan, there were (12.48±5.58) mSv, (9.30±2.99) mGy and (805.43±359.98) mGy. Those at chest abdomen-pelvic CT scan were (11.72±3.98) mSv, (10.82±2.53) mGy and (755.97±251.52) mGy.cm respectively. For lumbar CT, there were (12.12±2.32) mSv, (26.46±5.24) mGy and (787.00±149.37) mGy.cm respectively.

**Conclusion:**

the findings of this study shows that our values are slightly higher than those of developed countries. This first-ever CT practice evaluation at Agadir's Hassan II Hospital reinforced the need for further radiology training for computed tomography practitioners on parameters influencing image quality, dose, and protocols improvement.

## Introduction

The International Commission on Radiological Protection (ICRP) defines the principles of radiation protection as being the justification, optimization, and dose limitation [1]. The dose limitation principle does not apply to medical exposures to preserve the potential benefit of exposure; instead, the ICRP defines Diagnostic Reference Levels (DRLs) as a value to compare practices [2]. Thus, the International Basic Safety Standards (also known as BSS) require the establishment of DRLs to indicate the necessity for an inquiry to determine whether the values in practice are excessively high or unexpectedly low [3]. Such DRLs should be founded, as much as possible, on large-scale surveys that are relevant to the local circumstances. Moroccan radiological legislation lacks these requirements. Despite the need and widespread attention on this topic, research on large-scale nationwide surveys of patients during medical radiation exposure for assessing DRLs is primarily from Europe [4-17]. The Computed Tomography (CT) scans are a relatively high-dose imaging procedure that is known to contribute significantly to individual and community doses [18,19]. Therefore, the actions for practice and optimizing radiation protection of patients should be prioritized and implemented for the CT modality.

Despite the growing number of CT machines in Morocco, the Diagnostic Reference Levels (DRL) have yet to be defined. Thus, several previous studies have shown a significant variation in the doses administered to patients for the same radiological examination procedure [20-26]. The aim of this pilot study at Hassan II Hospital of Agadir was to estimate the doses during radiation medical exposure of four CT scan procedures so that local diagnostic reference levels (DRLs) could be established.

## Methods

A descriptive study in medical imaging was conducted at Hassan II Hospital in Agadir from September 2019 to January 2020. A general electric 16 CT with automatic exposure control system was used for all CT exams. The machine's quality controls were checked regularly, and all of the measurement parameters were within acceptable limits.

In this survey, 200 patients were considered for four CT imaging procedures, with 50 patients per localization. Patient-related parameters (age, clinical data, contrast media use), as well as X-ray exposure parameters, were collected. Other exposure-related parameters such as gantry tilt, tube current (mA), exposure time, tension (kV), slice thickness, number of slices, table increment, and the dose length product DLP (mGy.cm), as well as started and finished locations on the displayed CT dose index CTDI_vol_ (mGy), were taken into account. The International Commission on Radiological Protection (ICRP) conversion factor was used to evaluate the effective dose and DRL [9].

**Statistical analysis:** it was conducted by Microsoft Office Excel 2007. The arithmetic mean (also denoted as mean) and third quartile (75^th^ percentile) are used to express quantitative variables. Descriptive statistics were used to evaluate the CT data. Average CTDI_vol_ (mGy) for each sequence and total DLP per examination were calculated. The typical dose was estimated using median DLP and CTDI_vol_ (mGy) data. The rounded third quartile values of median distributions for CTDI_vol_ (mGy) and DLP (mGy.cm) data were used to set the DRL for Hassan II Hospital in Agadir. The results obtained were compared with those established by other countries.

## Results

Of the 200 CT scans collected, 25% were for the chest, 25% for the abdomino-pelvic, 25% for the chest-abdomen-pelvis, and 25% for the lumbar spine. The patients' average age was (48 ± 17) years (20-90 years), with 60% of them being 45 years old. The chosen patients' average weight was (67 ± 8) kg. All protocols were helical and had a voltage of 120 kV for the Acquisition parameters of the four procedures. With extremely varied acquisition parameters, the maximum charge (mAs) was used for chest abdomino pelvic and lumbar spine CT scans (350 mAs average) (Table 1).

**Table 1:** average acquisition parameters of the four most common CT-scan imaging in our service

Computed tomography- procedures	kV	mAs	Lenght of acquisition (cm)
**Chest**	120	163	42
**Abdomen pelvic**	120	183	78
**Chest abdomen pelvic**	120	350	69
**Lumbar spine**	120	350	29

**Patient-doses for four commonest CT-exams:** the results obtained show a variation in patient doses for the four most common CT examinations studied. The lumbar spine CT scan (mean 651 mGy.cm) showed the smallest dose variation (2-fold) while the greatest patient-dose variability was observed in the abdominal pelvic CT scan (8-fold) (Table 2).

**Table 2:** mean dosimetric parameters CTDI_vol_, DLP, and their ranges per localization studied

Computed tomography- procedures	CTDIvol (mGy)		DLP (mGy·cm)	
	Mean	Range	Mean	Range
**Chest**	9	5-17	375	169-686
**Abdomen pelvic**	6	4-11	517	138-1088
**Chest abdomen pelvic**	9	5-15	605	167-1035
**Lumbar spine**	22	15-28	651	345-845

**Comparison of DRLs obtained in this study with those adopted by other countries:** in Agadir's Hassan II hospital's radiology department, we were able to establish DRLs for chest, abdomino-pelvic, chest abdomino-pelvic, and lumbar spine CT-scans for adults depending on the 75^th^ percentile of the DLPs distribution in our sample (Table 3).

**Table 3:** comparison of the DRLs established in this study with those of other countries

	Our study DRLs		Belgium		China		Cameroun		France		UK	
			2020		2019		2017		2016		2014	
CT procedures	CTDI_vol_	DLP	CTDI_vol_	DLP	CTDI_vol_	DLP	CTDI_vol_	DLP	CTDI_vol_	DLP	CTDI_vol_	DLP
	11	428	8	260	8	285	22	715	10	350	13	440
**Chest**	9	805	10	570	-	-	15	716	13	650	15	745
**Abdomen pelvic**	11	756	8,5	800	-	-	-	-	12	800	20	1000
**Chest abdomen pelvic**	26	787	26	600	20	553	25	769	30	770	28	600

## Discussion

This descriptive study was carried out to propose DRLs for four of the most common adult CT-scan examinations at the radiology department of Hassan II hospital in Agadir. The 75^th^ percentile of DLP was 428 mGy.cm, 756 mGy.cm, 805 mGy.cm, and 787 mGy.cm for chest, chest abdomen pelvic, abdomino-pelvic, and lumbar spine CT-scans, respectively. Apart from the thoracic abdomino-pelvic procedure, our DRL values are superior to those established by other countries such as the United Kingdom (2014), France (2016), China (2019), and Belgium (2020) [4,27-29]. Furthermore, our DRLs for chest CT-scan, are lower than those in Cameroon (2017) [30]. Consequently, our patient-doses DRLs are between those of industrialized countries with advanced facilities and advanced cultures of optimization, such as China, the United Kingdom, France, and Belgium, and DRLs of an African country such as Cameroon. Looking forward to the development of a program to establish diagnostic reference levels at the national level, the DRLs obtained in this study can be used for optimization in Agadir's Hassan II Hospital. Radiologists should methodically add DLP notification on radiological reports for patient radioprotection reasons [31]. On lumbar spine CT scans, higher DRLs have been seen in Belgium, China, the United Kingdom, France, and Cameroon [4,27-30]; this is due to the presence of multiple high attenuating bone structures in the spine, which necessitates the use of large doses to provide satisfactory image quality. We have evaluated the first results of DRLs, which can already be used for optimization while we wait for a national survey to assess the DRLs with the help of competent authorities. In Morocco, further research is needed to establish DRLs for pediatric CT as well as for procedures of all radiological modalities.

## Conclusion

The results obtained show that DLP values of 428 mGycm, 756 mGy.cm, 805 mGy.cm, and 787 mGy.cm for chest, chest abdomen pelvic, abdomino-pelvic, and lumbar spine CT-scans, respectively, can be utilized as diagnostic reference levels in CT imaging in adults in Agadir's Hassan II hospital's radiology department. These DRLs are slightly higher than those of some developed countries. Hence the need for a periodic continuous training program on the parameters influencing the quality of the images, the dose, and the improvement of the protocols for the practitioners.

### What is known about this topic

- *Computed tomography scan exposures data are not yet investigated at this center;*
- *The radiation doses for CT scan procedures are not yet documented;*
- *Diagnostic reference levels data are not yet established for CT scan procedures*.

### What this study adds

- *The DRLs established will guide the process of optimization;*
- *The variations in the radiation dose during CT scan practice will be limited;*
- *This is the first assessment of DRLs in our radiology department*.
